# Prevalence of musculoskeletal disorders amongst flower farm workers in Kenya

**DOI:** 10.4102/sajp.v77i1.1515

**Published:** 2021-03-09

**Authors:** Jotham M. Munala, Benita Olivier, Wallace M. Karuguti, Simon M. Karanja

**Affiliations:** 1 Department of Physiotherapy, Faculty of Rehabilitative Sciences, Kenya Medical Training College, Nairobi, Kenya; 2 Department of Rehabilitation, School of Medicine, Jomo Kenyatta University of Agriculture and Technology, Nairobi, Kenya; 3 Department of Physiotherapy, Faculty of Health Sciences, University of the Witwatersrand, Johannesburg, South Arica; 4 Department of Physiotherapy, School of Medicine, Jomo Kenyatta University of Agriculture and Technology, Nairobi, Kenya; 5 Department of Public Health, Faculty of Public Health, Jomo Kenyatta University of Agriculture and Technology, Nairobi, Kenya

**Keywords:** work-related musculoskeletal disorders, farm workers, prevalence, musculoskeletal pain, chronic pain

## Abstract

**Background:**

Work-related musculoskeletal disorders (WMSDs) are a global public concern for health and social-care systems, as well as individuals. They are the second-most prevalent cause of disability globally.

**Objectives:**

The primary objective was to determine the prevalence of WMSDs amongst flower farm workers. The secondary objective was to determine the association between the socio-demographic characteristics and the presence of WMSDs in the previous 12-month period.

**Method:**

A cross-sectional descriptive study was conducted. A sample of 270 participants was drawn from 897 farm workers. Quantitative data related to musculoskeletal disorders were collected using the Nordic Musculoskeletal Questionnaire (NMQ). Descriptive statistics were undertaken using frequencies and percentages. Inferential statistics were analysed using a chi-squared test (X^2^) based on an alpha level of *p* < 0.05.

**Results:**

A total of 184 (68.1%) respondents reported musculoskeletal discomfort. Amongst the 184 respondents, 178 were performing general farm work. Most 103 (38.1%) of the WMSDs were reported in the lower back. There was a strong association between job designation as a general worker (*p* = 0.016), an older age (*p* = 0.027) and having worked for a long time as a farm worker (*p* = 0.041) and WMSDs.

**Conclusion:**

Flower farm workers in Kenya were found to be heavily burdened by WMSDs. Furthermore, the job designation, older age, as well as having worked for a long time, predisposes workers to the risk of developing WMSDs.

**Clinical implications:**

The high prevalence of WMSDs necessitates policy reform in the flower farm industry.

## Introduction

Work-related musculoskeletal disorders (WMSDs) are the second most prevalent cause of disability in both developed and developing countries (Shuai et al. 2014). Work-related musculoskeletal disorders place a considerable burden on healthcare systems, individuals and social-care systems (Woolf & Pfleger 2003), incurring substantial costs for public health systems (Darwish & Al-Zuhair 2013).

Work-related musculoskeletal disorders are classified as an occupational illness as they are more prevalent amongst the working class than the general population (Damayanti, Zorem & Pankaj 2017; Vedovato & Monteiro 2014). Most WMSDs develop over a period of time and are caused by the work itself and its repetitive nature involving either low or high-intensity loads (Shafti et al. 2016; Shafti et al. 2016). The structures affected are neuromusculoskeletal (Ganiyu et al. 2015; Nunes & Bush 2012) and the general symptoms of WMSDs vary from dull discomfort to pain and in time, reduced function (Shafti et al. 2016).

According to Yassi (2000), statistics from the US Bureau of Labor show that WMSD syndrome accounts for about 65% of occupational disorders and that carpal tunnel syndrome is the leading work-related condition. In China, the overall 1-year prevalence level is 85.5% with the shoulder, neck and lower back scoring 62%, 60.3% and 54.3%, respectively, as the most affected body regions (Wang et al. 2017). A study amongst female Saudi school teachers report that the low back was the most affected area at 38.1%, followed by the knee, heel and shoulder at 26.3%, 24.1% and 20.6%, respectively (Abdulmonem et al. 2014). Wanyonyi and Frantz (2015), reported a relatively higher 1-year prevalence of WMSDs ranging between 15% and 93.6% in their systematic review.

In a WMSD study on butchers in the Kano metropolis in Nigeria, point and 1-year prevalence levels are 74.5% and 88.2%, respectively (Kaka et al. 2016) and in Ibadan, Nigeria, a 1-year prevalence rate of WMSD amongst occupational drivers is 89.3% (Akinpelu et al. 2011). A study on musculoskeletal pain in women working at small-scale farming activities in South Africa showed a 1-year prevalence range of 63.9% – 73.3% (Naidoo et al. 2009). Seventy per cent of the workers complained of WMSD at the Apparel Assembly Plant in Uganda in three different body areas (Tebyetekerwa, Akankwasa & Marriam 2017). Furthermore, a systematic review of 24 studies on the prevalence of WMSD amongst farmers showed a 1-year prevalence of 76.9%, with a lifetime prevalence of 90.6% (Osborne et al. 2012).

There is a close relationship between specific occupations and the development of WMSDs. For instance, manual lifting, acquired awkward postures, prolonged static postures, labour-intensive tasks, and the working environment may lead to WMSDs (Azim 2016; Ganiyu et al. 2015). Other socio-demographic characteristics, such as age, gender and the period of time that an individual has worked (Health and Safety Executive [HSE] 2019; Shuai et al. 2014; Singh & Arora 2010) have shown a relationship with the development of WMSDs. On an average, females had higher levels of WMSD’s in the Uganda apparel firms, and this is probably because of the numerous responsibilities they had (Tebyetekerwa et al. 2017).

Although many tools have been developed with the sole intention of making work easier and tools more user-friendly, there are still many challenges that lead to WMSDs (Shafti et al. 2016). Shafti et al. (2016) further highlight the fact that, white-collar occupations such as teaching and banking also expose individuals to the risk of WMSDs. Workers in agriculture, forestry, construction, human health (HSE 2019), transport and storage are, however, affected to a greater extent (Singh & Arora 2010).

In agriculture, it is noted that both farm workers and experienced farmers perceive WMSDs as a ‘normal’ experience and inevitable on account of the type of work performed (Singh & Arora 2010). In Kenya, no studies have been conducted on WMSDs in horticulture, yet farming activities dominate the economic sector. Most farmers and farm workers are deployed in a very competitive environment and are under great pressure to produce in large quantities. As such, they are more exposed to WMSDs. As noted by Shafti et al. (2016), although many tools have been developed to reduce the risk of developing WMSDs, there are still many challenges in the usage of these tools, including their application in areas such as planting, picking of ready flowers, and general crop and farm management.

Therefore, the primary objective of our study was to determine the prevalence of WMSD amongst flower farm workers in Kenya. The secondary objective was to determine the association between the presence of WMSD over the previous 12 months.

## Methods

A cross-sectional study included 897 workers on a flower farm that grows flowers for export. Farm work is executed manually from day-to-day, and includes long forceful physical stresses. Farm workers are divided into two categories: sprayers who get a few days’ on-job training on spraying-techniques at various periods of growth, and general workers who carry out the rest of the manual tasks on a rotational basis such as planting, weeding, picking flowers, transporting flowers from the farm and general farm maintenance duties.

The formula for determining the sample size, namely *n* = Z^2^pq/d^2^, developed by Cochran (1977) was used, and the calculated sample size was 270 farm workers. A systematic sampling method was used to select the individual farm workers from an alphabetical list of all farm workers.

All farm workers aged 18 years and above were invited to participate. Farm workers who had worked for at least 4 months in horticultural farming were included in order to more accurately determine an association between exposure to farming and developing musculoskeletal disorders. Farm workers who were pregnant or who had joined the horticultural farming industry with pre-existing musculoskeletal disorders were excluded.

### Data collection tool

The Nordic Musculoskeletal Questionnaire (NMQ) was used to collect data. The Nordic Musculoskeletal Questionnaire is a standardised questionnaire that is cross-culturally adapted and used in the screening of musculoskeletal discomfort (Crawford 2007). Crawford (2007) established that the sensitivity and specificity of NMQ ranged from 66% to 92%, and 71% to 88%, respectively. All of the respondents completed the questionnaire as to whether they had experienced any musculoskeletal discomfort over the previous 12 months. The socio-demographic characteristics that were obtained included job designation, gender, age, weight and the length of time the farm worker had been working at the time of our study.

Written informed consent was sought from the farm workers before they were included in our study.

Data collection was carried out over a period of 2 weeks in December 2019. An information sheet was given to each of the 897 farm workers. The first author generated an extensive list of 897 farm workers on the farm. As the sample size was 270, every third farm worker was selected according to a number ranging between one and 270, until the number 270 was reached.

All the farm workers present during a particular work day shift were briefed about our study on each visit by the authors. Thirty-three (33) trained research assistants explained the information individually to the farm workers who met the inclusion criteria and helped them to understand, complete and sign the consent form. Research assistants took each participant through the NMQ and helped participants to complete the NMQ, which took between 3 min and 5 min. The first author then reviewed each NMQ and ensured that all the relevant sections in the questionnaire had been completed.

### Data analysis and management

Quantitative data from the NMQ was keyed into a Microsoft Excel spreadsheet database and secured using passwords known only to the first author.

Statistical analysis was undertaken using Statistical Package for the Social Sciences (SPSS) version 25. Frequencies and percentages were used to describe the data. The associations between the presence of WMSDs and variables (job position, gender, age, weight and length of duration worked) were then analysed using Pearson’s chi-squared test (X^2^) , based on an alpha level of *p* < 0.05.

### Ethical consideration

The authors acquired permission and ethical clearance from Jomo Kenyatta University of Agriculture and Technology (reference number: JKU/2/4/896B) and the Farm Director through the Director of Human Resources and Administration.

## Results

### Prevalence of musculoskeletal disorders

A total of 270 farm workers – 30% of the total population – participated in our study. The majority 184 (68.1%), reported having experienced discomfort in either one or more of their body areas over the previous 1 year. Most WMSDs were reported in the lower back (38.1%), followed by the wrist and hand (24.1%) and the ankle and feet (24.1%) as shown in Table 1.

**TABLE 1:** Prevalence of musculoskeletal disorders according to body area (*n* = 270).

	Frequency (*n*)	Percentage (%)
**Prevalence of work-related musculoskeletal disorders**		
Yes	184	68.1
No	86	31.9
**Prevalence according to body parts**		
Neck discomfort		
Yes	51	18.9
No	219	81.1
Shoulder discomfort		
Yes	57	21.1
No	213	78.9
Elbow discomfort		
Yes	49	18.1
No	221	81.9
Wrist and hand discomfort		
Yes	65	24.1
No	205	75.9
Upper back discomfort		
Yes	58	21.5
No	212	78.5
Lower back discomfort		
Yes	103	38.1
No	167	61.9
Hip and thigh discomfort		
Yes	52	19.3
No	218	80.7
Knee discomfort		
Yes	51	18.9
No	219	81.1
Ankle and feet discomfort		
Yes	65	24.1
No	205	75.9

### Relationship between musculoskeletal disorders and socio-demographic characteristics

Respondents designated as general workers were the most affected, with 178 (70%) having experienced musculoskeletal disorders in the previous 12-month period (*p* = 0.016). The majority 98 (76%) of the participants in the 40–49 year age group experienced WMSDs. Additionally, 53 (80%) of those who had worked for 11–15 years experienced WMSDs, as shown in Table 2.

**TABLE 2:** Relationship between work-related musculoskeletal disorders and socio-demographic characteristics.

Socio-demographic characteristics	WMSDs *n* = 184		No WMSDs *n* = 86		Total *n* = 270		*p*
	*n*	%	*n*	%	*n*	%	
Job position							*0.016
General worker	178	70	77	30	255	94	
Sprayer	6	40	9	60	15	6	
Gender							0.165
Male	86	64	48	36	134	49.6	
Female	98	72	38	28	136	50.4	
Age (Years)							*0.027
<30	11	48	12	52	23	9	
30–39	62	63	36	37	98	36	
40–49	98	76	31	24	129	48	
≥50	13	65	7	35	20	7	
Weight (Kg)							0.502
40–60	62	74	22	26	84	31	
61–80	114	66	58	34	172	64	
81–100	7	58	5	42	12	4	
>100	1	50	1	50	2	1	
Length of time in worked (Years)							*0.041
≤5	31	53	27	47	58	21	
6–10	78	69	35	31	113	42	
11–15	53	80	13	20	66	24	
16–20	14	67	7	33	21	8	
21–25	6	60	4	40	10	4	
≥26	2	100	0	0	2	1	

* , *p*-value below 0.05.

Note: An asterisk is used to highlight an area of interest, where the p-values are significant (job position, age and duration one had worked).

WMSD, work-related musculoskeletal disorders.

## Discussion

This is the first study on WMSDs in horticultural farming in Kenya and is important in a country where agricultural activities dominate the economic sector.

The 1-year prevalence of WMSD was notably high (68.1%). This was probably because of the laborious nature of work that the farm workers are exposed to. Flower farming is labour intensive in nature and the product is perishable, so it demands both a high pace of work and often working overtime. The overall duties involve repeated manual activities undertaken in a difficult posture.

In South Africa, a cross-sectional study on musculoskeletal pain in 911 women involved in small-scale agricultural activities with the same study design had similar results with a 67% (*n* = 574) 1-year prevalence of chronic musculoskeletal pain (Naidoo et al. 2009). Both studies focused on farm workers even though Naidoo et al. (2009) only included females.

In Uganda, even though the study focused on a sector of the economy that differed from farming, a cross-sectional study on 103 workers in the Apparel Assembly Plant reported a 1-year prevalence rate of 68.9% (Tebyetekerwa et al. 2017); there again, the data were collected using the NMQ. Similarities in the findings suggest that WMSDs generally occur at a higher frequency level in a working population. The prevalence in our study was lower than that for a study in Ibadan, Nigeria, where a 1-year prevalence rate of WMSD amongst occupational drivers was 89.3% (Akinpelu et al. 2011), whilst a WMSD study on butchers in Kano metropolis in Nigeria reported a point prevalence of 74.5% (Kaka et al. 2016). The difference between the prevalence found in our study (68.1%) from that found by Kaka et al. (2016), indicate that the nature of the work environment may play a significant role in the prevalence of WMSDs.

Most of the WMSD cases were reported in the low back, this was most likely because of the occupational strain that the low back is subjected to during the day-to-day work tasks. In addition, the low back in this case acted as leverage in transition of the body weight across these postures. The second and third most affected body areas were the wrists and hands, and then the ankles and feet, probably because farm workers are involved in activities that are repetitive and manual in nature such as planting, spraying, sweeping, transporting flowers, general farm maintenance and picking of flowers in a stooped or prolonged standing positions.

A systematic review of 24 studies on the prevalence of WMSD amongst farmers reported that the lower back was the most affected region, with a 1-year prevalence of 47.8%, followed by the upper limb in a range of 3.6% – 71.4% (Osborne et al. 2012). In a WMSD study in China amongst medical staff (*n* = 1017), results showed that the most severely affected body areas are the shoulder joint, neck and back, with a prevalence of 62%, 60.3% and 54.3%, respectively (Wang et al. 2017). In a WMSD study amongst farmers in Kanpur, India, low backache was common, with 69%, followed by the knee, shoulder and neck pain, with a prevalence of 39%, 22% and 10%, respectively (Gupta 2013). In Irish farmers (*n* = 103), low back pain was the most common region affected, with a 31% prevalence (Osborne et al. 2013).

The farm workers designated as general workers were the most severely affected. This is because of the nature of their work that is repetitive and entails prolonged working hours in static, standing postures or stooping postures and lifting of loads. The general workers were not trained to accomplish their work, probably because of the assumption that it is a general duty, needing less training input. It is important to note that even though the general workers had a higher prevalence of WMSDs compared to the sprayers, the results should be interpreted with caution as the group of sprayers was smaller in number.

The higher number of females who suffered from WMSDs compared to males may be as a result of their overall reduced physical capacities. These findings are similar to those of Tebyetekerwa et al. (2017) and Xiao et al. (2013).

There was an association between increased age and the development of WMSDs. This probably suggests prolonged exposure to a hazardous physical environment, and laborious and repetitive body movements. Our results are similar to those reported by HSE (2019) on WMSDs and by Australia Safe Work (2016).

The period which a farm worker had worked emerged as a significant factor associated with the development of WMSDs. This may have been because of the lengthy day-to-day exposure to forceful physical stresses. The findings are similar to those of Australian Safe Work (2016).

## Limitations of our study

Our study employed a cross-sectional study design and causality between the presence of WRMSDs and the factors described could not be determined. Therefore, assumptions are limited to associations.

Another shortcoming of our study is that it captured very few of the sprayers (*n* = 15) as opposed to the general workers (*n* = 255). As such, the sprayers might not have been well represented for the purposes of the sub-group analysis.

## Conclusion

It is no doubt that farm workers are heavily burdened by WMSDs. In addition, based on the associations determined in our study, specific farm job designations, the age of the worker, and the duration of time involved over a long period may predispose workers to various risks that may result in the development of WMSDs.

We recommend that future researchers need to assess the time lost by the workers during the periods when they are off sick and/or hospitalised. Consequently, future researchers need to capture the costs incurred by the farm workers in seeking medical attention. Lastly, with body mass index (BMI) being a hypothetical socio-demographic characteristic in the development of a WMSD, future researchers need to incorporate measurements to effectively determine the effect of BMIs on WMSDs.
